# Supplementary material for: Activity-Guided Proteomic Profiling of Proteasomes Uncovers a Variety of Active (and Inactive) Proteasome Species
Source: Mol Cell Proteomics. 2024 Jan 29;23(3):100728. doi: 10.1016/j.mcpro.2024.100728 (PMC10907802; doi:10.1016/j.mcpro.2024.100728)
Supplement: Supplemental Data [file mmc1.docx]

**Supplemental information**

**Activity-Guided Proteomic Profiling of Proteasomes Uncovers a Variety of Active (And Inactive) Proteasome Species**

Manisha Priyadarsini Sahoo^1,*^, Tali Lavy^1,*^, Noam Cohen^1^, Indrajit Sahu^1,#^ and Oded Kleifeld^1^

^1^Faculty of Biology, Technion-Israel Institute of Technology, Technion City, Haifa 3200003, Israel.

# Current address: Division of Medical Research, Faculty of Medical & Health Sciences, SRM University, Chennai 603203, India

* These authors contributed equally to the manuscript.

**List of items:**

Figure S1: Proteomic analysis of whole cell extracts.

Figure S2: Proteasome peptidase activity assay.

Figure S3:

Figure S4: Comparison of complexes ∆α3.

Figure S5: Comparison of complexes ∆Sem1.

Figure S6: Comparison of 26S to Lidess 26S in ∆Sem1.

Figure S7: Proteasomes and Fub1 amounts in different strains

Figure S8: Representative MS/MS spectra of matured β1, β2 and β5 signature peptides

Figure S9: Representative MS/MS spectra of immatured β2 and β5 signature peptides

Table S1: Whole cell proteomics - Provided in attached Excel file.

Table S2: Whole cell proteomics - Go enrichment analysis of proteins with significant abundance changes - Provided in attached Excel file.

Table S3: Activity-guided profiling of active proteasome complexes (entire lane) - Provided in attached Excel file.

Table S4: Comparison of native and denaturing extraction of WT proteome - Provided in attached Excel file.

Table S5: Comparison of WT whole proteome analysis and activity-guided profiling - Provided in attached Excel file.

Table S6: Activity-guided profiling of wt active proteasome complexes - Provided in attached Excel file.

Table S7: Activity-guided profiling of ∆α3 active proteasome complexes - Provided in attached Excel file.

Table S8: Activity-guided profiling of ∆Sem1 active proteasome complexes - Provided in attached Excel file.

Table S9: Distribution of proteasome complexes based on peptidase activity (Quantified by ImageJ) - Provided in attached Excel file.

Table S10: Activity-guided profiling of all active proteasome complexes (separate complexes)- was used to determine the PIPs distribution across proteasome complexes in each strain - Provided in attached Excel file.

Table S11: Semi-tryptic peptides search of Activity-guided profiling of all active proteasome complexes (separate complexes) - was used to determine the activation state of the proteolytic subunits across proteasome complexes in each strain - Provided in attached Excel file.


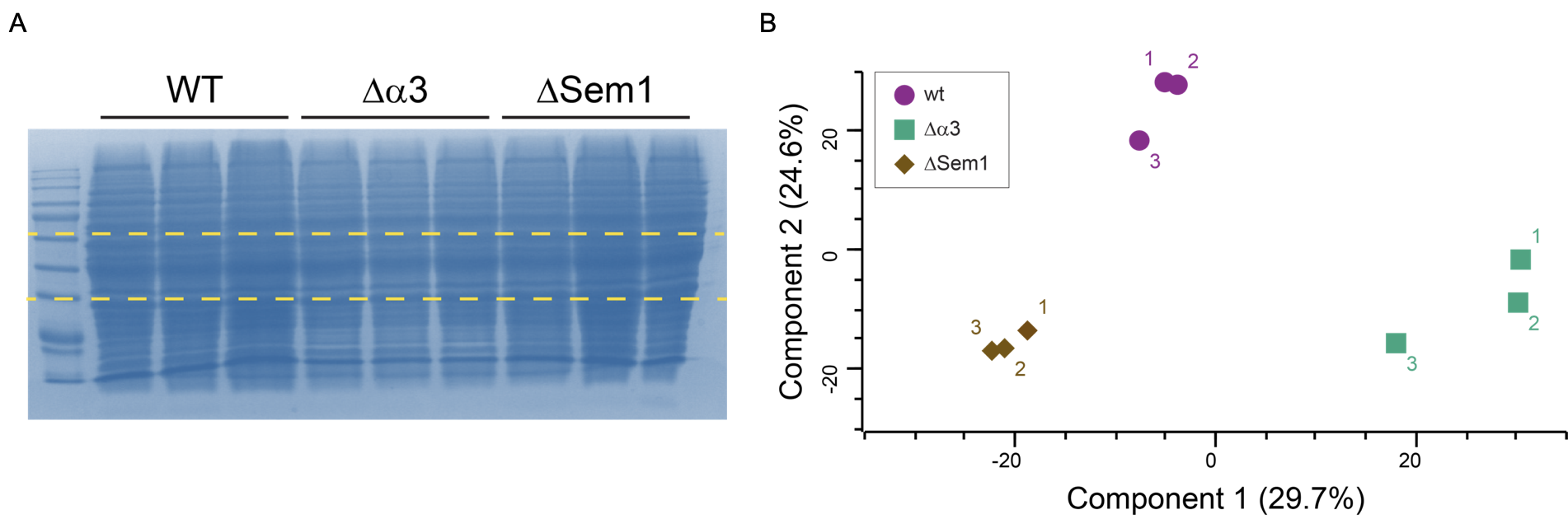


**Figure S1: Proteomic analysis of whole cell extracts. A**. Sample preparation for whole cell proteomics was done following SDS-PAGE separation of whole cell lysate of WT, ∆α3 and ∆Sem1 cultures. Each lane was cut into 3 regions that were subject to in-gel tryptic digestion**. B.**  PCA analysis of the LFQ data of identified proteins obtained from the three replicates of each strain.


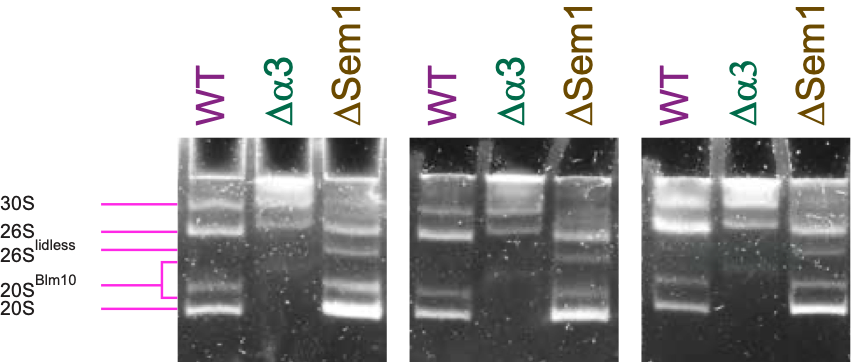


**Figure S2: Proteasome peptidase activity assay** Whole cell native lysate of WT, ∆α3 and ∆Sem1 cultures were loaded and separated by native-PAGE. Peptidase activity was monitored using the proteasome substrate LLVY-AMC (Elsasser S. et al., 2005).

**
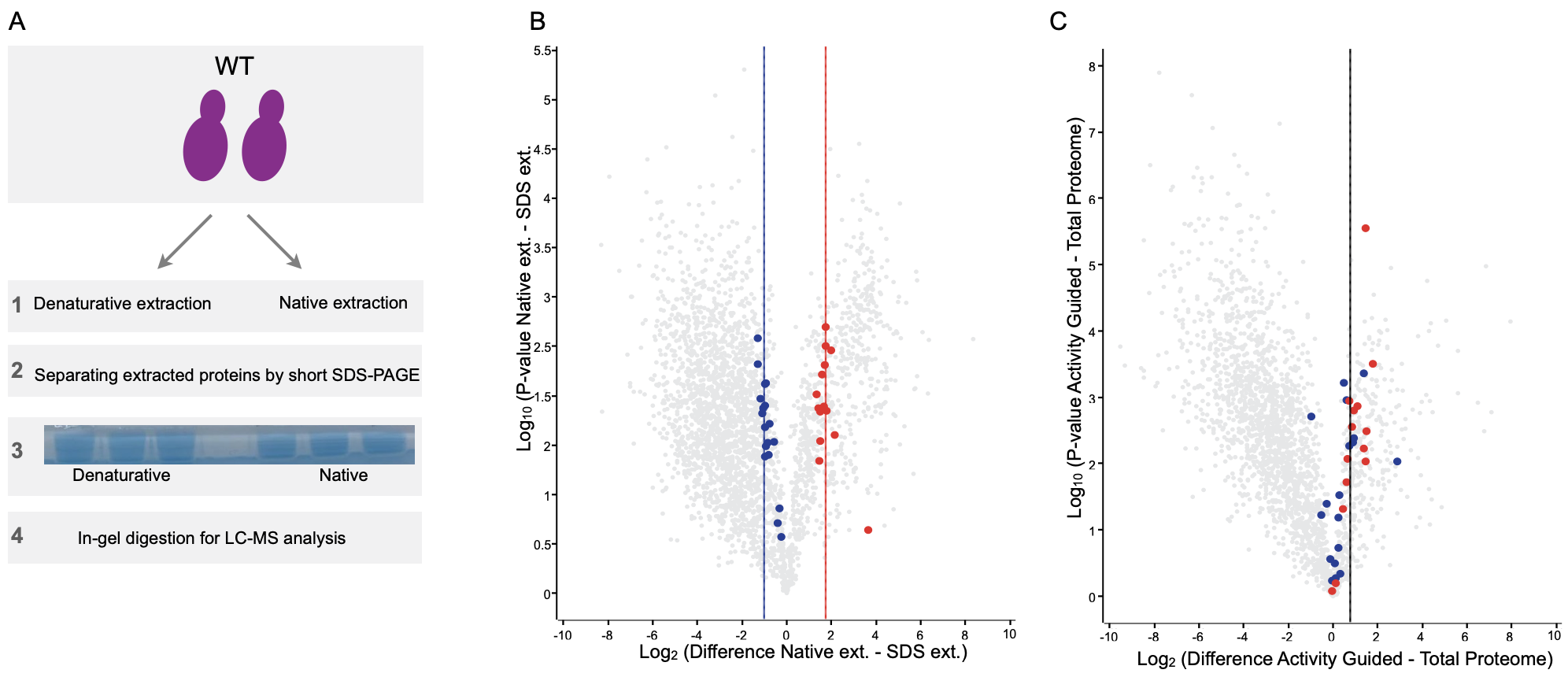
Figure S3: Impact of extraction conditions on proteasomal subunits identification. A.** Comparison Workflow: A culture of the WT strain was divided into two aliquots. Proteins from one aliquot were extracted under denaturing conditions with SDS, while those from the other were extracted under native conditions, like those used in activity-guided proteasome profiling studies. Both samples were then loaded and briefly separated using SDS-PAGE. Each entire lane from the gel was subsequently subjected to in-gel tryptic digestion followed by LC-MS/MS analysis. **B.** Abundance changes in WT proteasomal subunits following protein extraction under native and denaturing conditions. The 20S subunits are indicated in red, and the 19S subunits in blue. The red and blue lines represent the average changes in the 20S and 19S subunits, respectively. **C.** Abundance changes of WT proteasomal subunits between whole proteome samples (extracted with SDS under denaturing conditions) and activity-guided proteasome profiling samples (extracted under native conditions). The 20S subunits are indicated in red, and the 19S subunits in blue. The grey line represents the average change of all subunits.


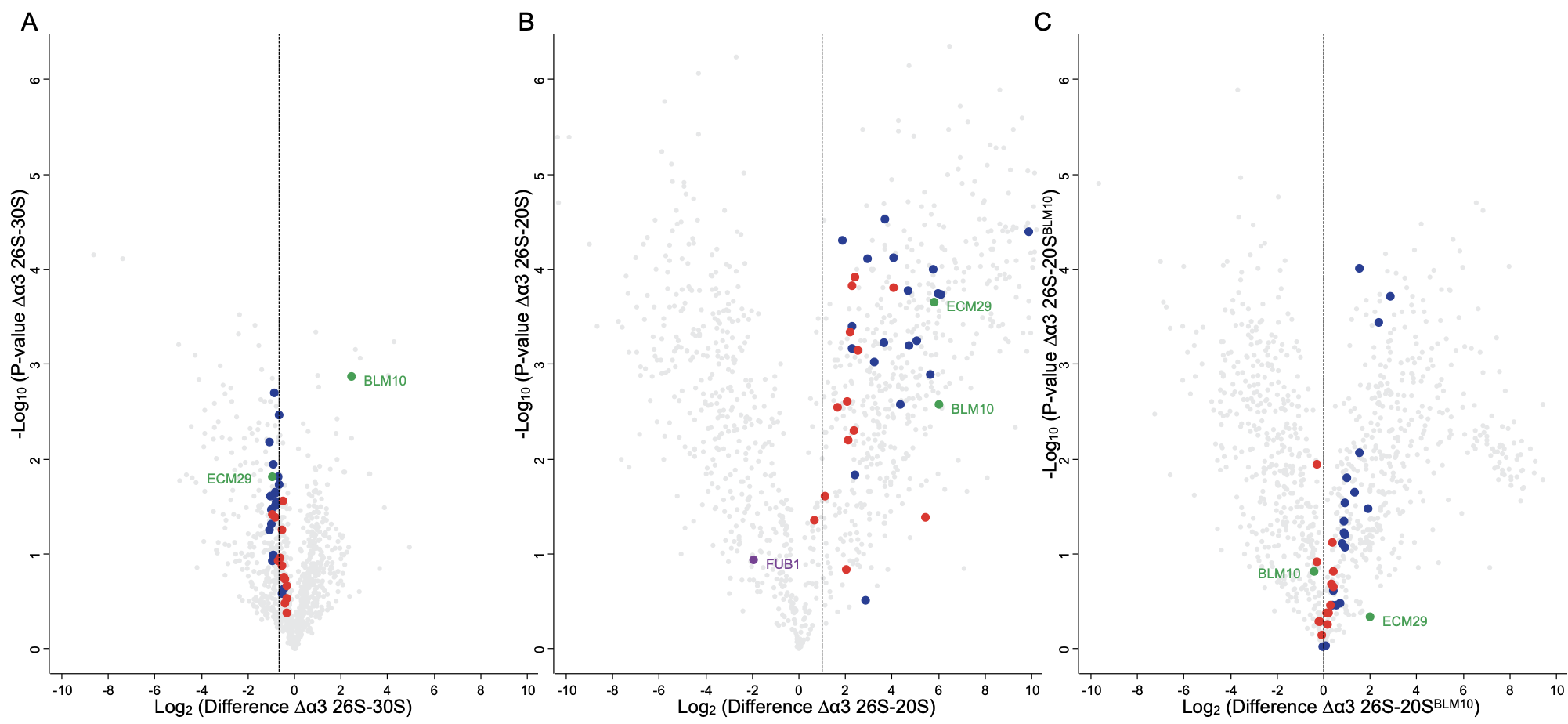
**Figure S4: Comparisons of complexes ∆α3 strain.** **A.** Changes in proteasome subunits and PIPs content of ∆α3 26S to 30S complexes. **B.** Changes in proteasome subunits and PIPs content of ∆α3 26S to 30S complexes. **C.** Changes in proteasome subunits and PIPs content of ∆α3 26S to 26S^Blm10^ complexes.


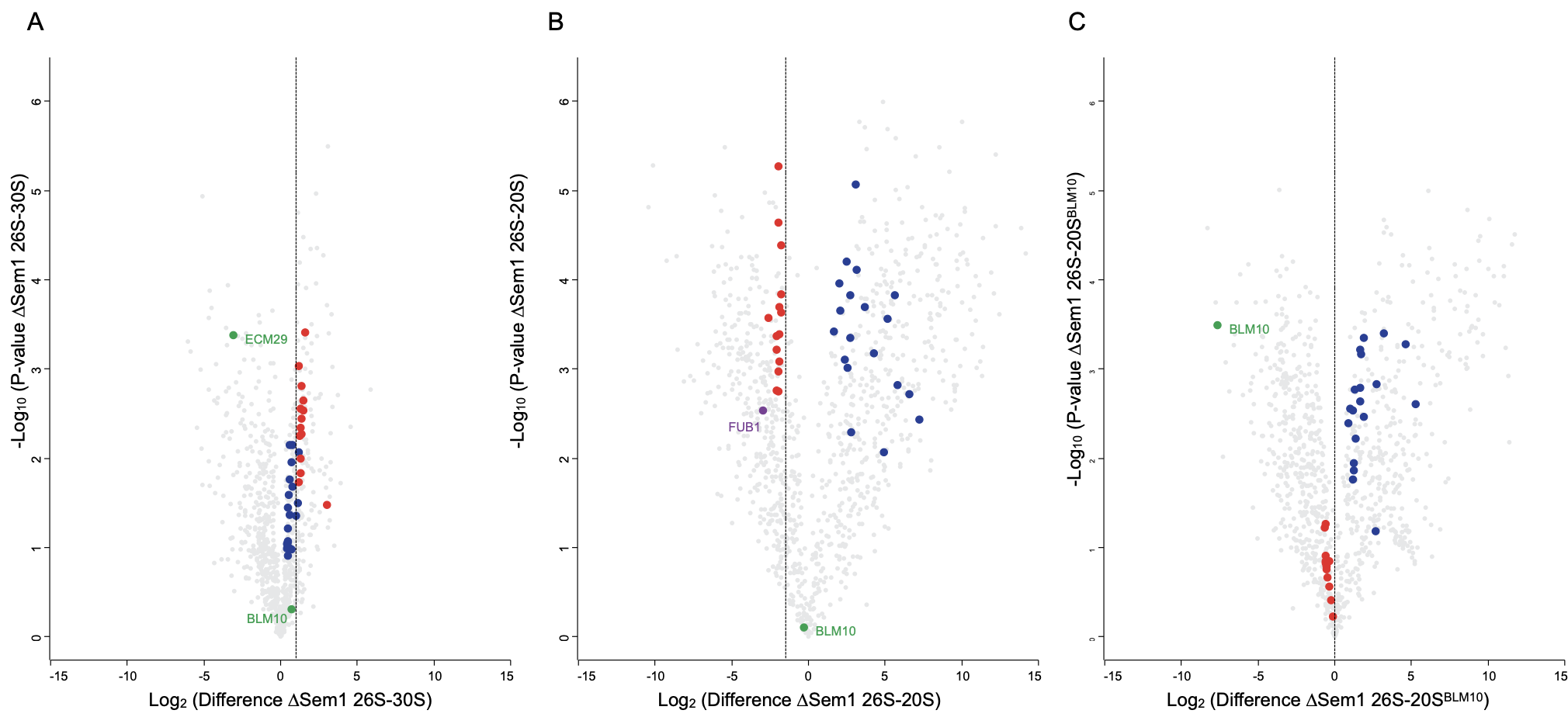
**Figure S5: Comparison of complexes of ∆Sem1 strain**. **A.** Changes in proteasome subunits and PIPs content of ∆Sem1 26S to 30S complexes. **B.** Changes in proteasome subunits and PIPs content of ∆Sem1 26S to 30S complexes. **C.** Changes in proteasome subunits and PIPs content of ∆Sem1 26S to 26S^Blm10^ complexes.


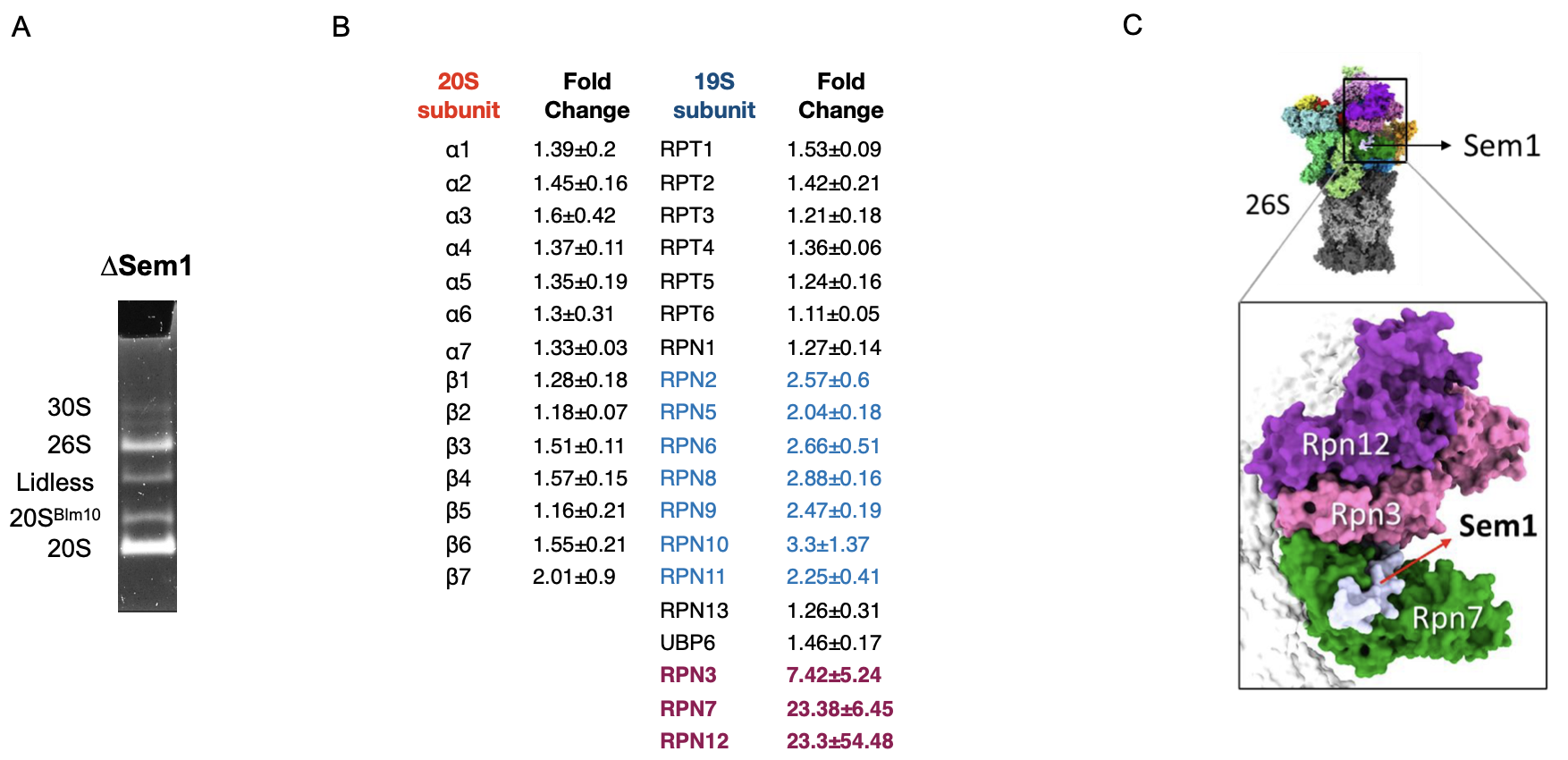


**Figure S6: Comparison of 26S to Lidess 26S in ∆Sem1. A.** Native gel shows active proteasome bands from ΔSem1 strain. **B.** The relative fold change of all individual 20S and 19S subunits in 26S proteasome compared to the Lidless-26S proteasome. **C.** Sem1 position in 26S proteasome structure shows direct interactions with Rpn3 and Rpn7 subunits and the proximity to Rpn12. The 26S presentation was generated with ChimeraX (Pettersen E.F. et al., 2021) based on Yeast proteasome structure PDB code: 6J2X (Ding Z., et al. 2019).

**
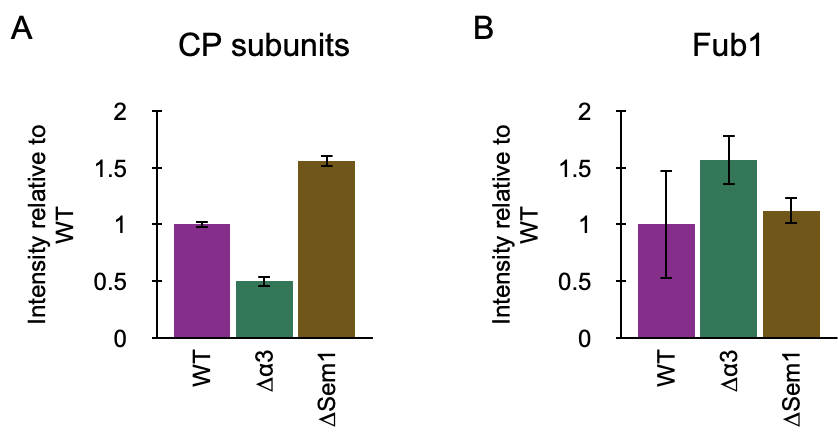
**

**Figure S7: Proteasomes and Fub1 amounts in different strains . A.** Comparison of the total amounts of proteasomes in the different strains. The LFQ intensities of all CP subunits were summed, and the WT total intensity was set as 1 and used as a reference to the comparison to the other strains. **B.** Comparison of Fub1 amount in the different strains. The LFQ intensity of Fub1 in WT was set as 1 and used as a reference to the comparison to the other strains. Note: WT Fub1 LFQ intensities were calculated only in 2 repeats out of 3.

**
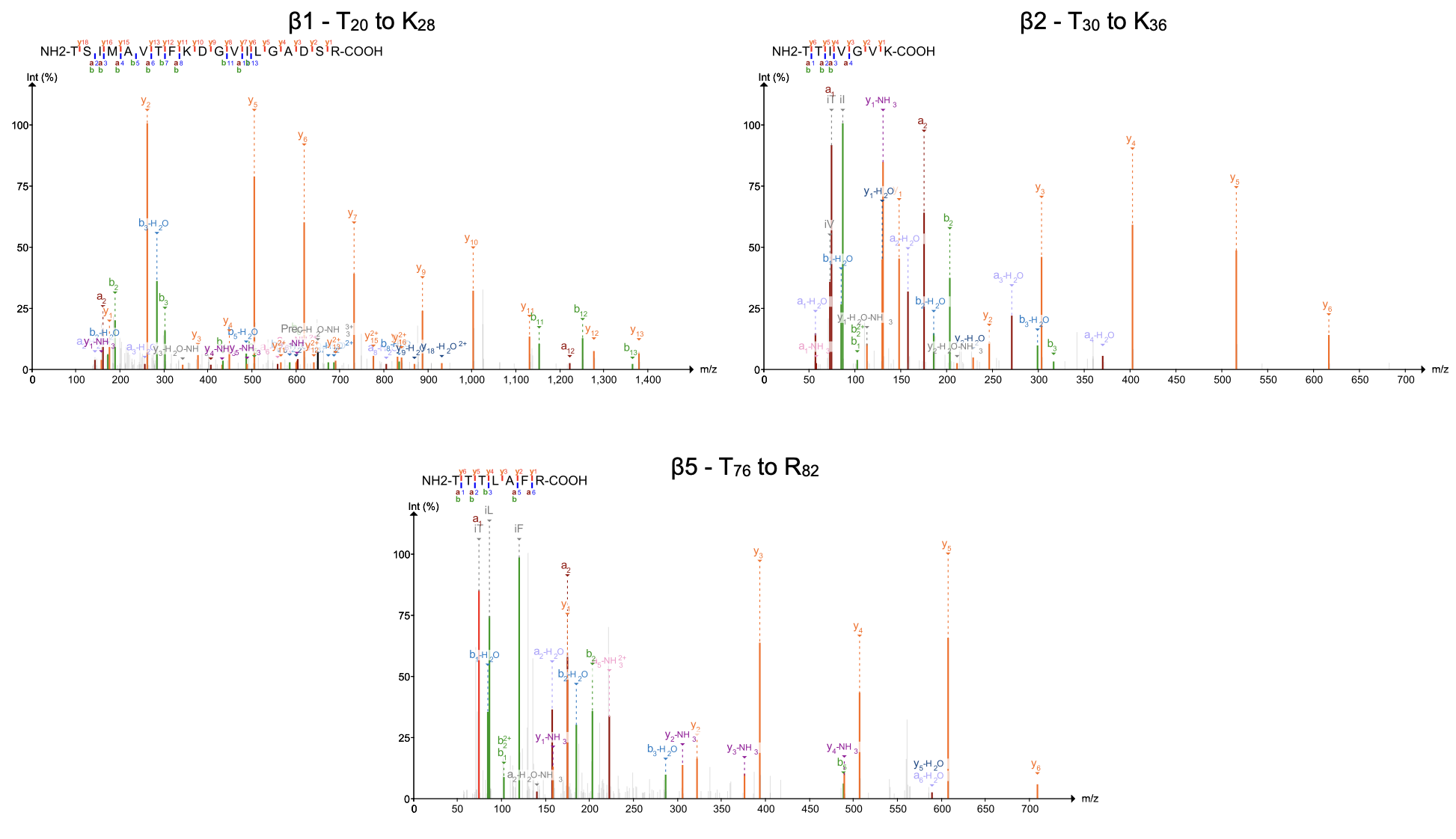
**

**Figure S8: Representative MS/MS spectra of matured β1, β2 and β5 signature peptides.**

**
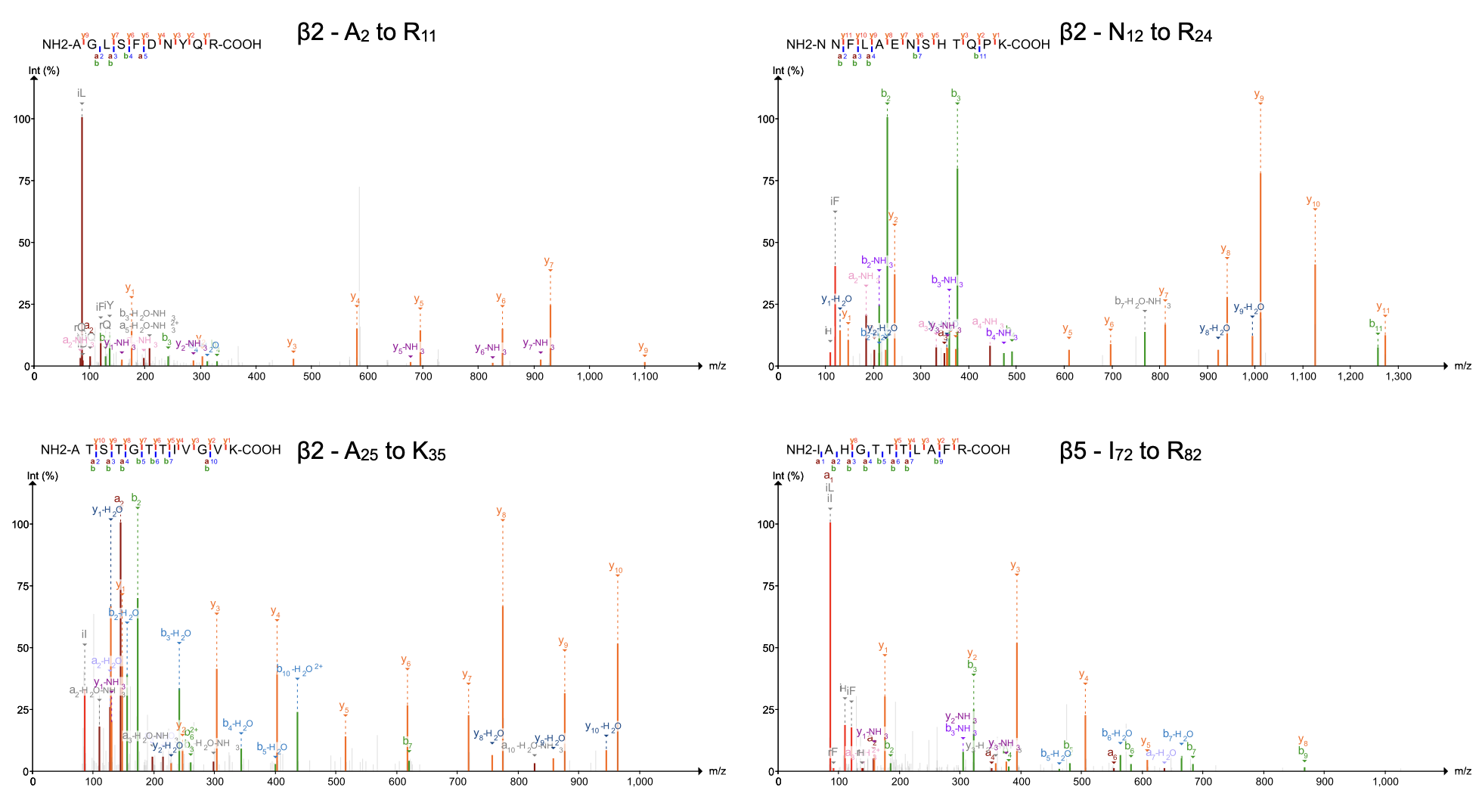
**

**Figure S9: Representative MS/MS spectra of non-matured β2 and β5 signature peptides.**

References:

S. Elsasser, M. Schmidt, D. Finley, Characterization of the proteasome using native gel electrophoresis. *Methods in Enzymology* **398**, 353–363 (2005).

E. F. Pettersen, *et al.*, UCSF ChimeraX: Structure visualization for researchers, educators, and developers. *Protein Sci* **30**, 70–82 (2021).

Z. Ding, *et al.*, Structural Snapshots of 26S Proteasome Reveal Tetraubiquitin-Induced Conformations. *Mol Cell* **73**, 1150-1161.e6 (2019).
